# How do parasites and predators choose their victim? A trade‐off between quality and vulnerability across antagonistic interactions

**DOI:** 10.1111/brv.70037

**Published:** 2025-05-14

**Authors:** Mairenn C. Attwood

**Affiliations:** ^1^ Department of Zoology University of Cambridge Downing St Cambridge CB2 3EJ UK; ^2^ FitzPatrick Institute of African Ornithology University of Cape Town Rondebosch Cape Town 7701 South Africa

**Keywords:** trade‐off, host choice, parasite, predator, prey, quality, susceptibility, vulnerability, antagonist

## Abstract

From blood‐sucking lice and food‐stealing gulls to pandemic‐inducing viruses and egg‐eating snakes: parasites and predators are ubiquitous in shaping ecology and evolution. Fundamental to these interactions is the way in which parasites and predators choose their victim. Here, I argue that a trade‐off between host quality and vulnerability can be generalised across systems to understand parasites' choice of hosts. This principle defines quality as the value of resources a host has, and vulnerability as the ease with which a parasite can obtain those resources. A parasite can choose a low‐quality host, which is easier to attack but offers limited resources, or a high‐quality host, which is more challenging to attack but offers more resources if the parasite is successful. The optimal decision for a parasite will depend on its ecology and the shape of the trade‐off in a given system. The trade‐off applies equally to predator–prey systems. Many studies of different types of parasitism and predation across taxa have investigated traits pertaining to quality or vulnerability, but their findings have not previously been integrated. Doing so makes it possible to draw out broad principles that determine whether quality or vulnerability has the greater impact on victim choice. It can also help explain contradictory findings, such as why the same antagonists choose high‐quality victims in some studies, and low‐quality victims in others. Further applications include predicting the effects of global change on host–parasite and predator–prey dynamics, and providing an integrated perspective on coevolutionary adaptations.

## I. INTRODUCTION

How do parasites choose their host? We can imagine parasites choosing a strong, fit host, which provides excellent resources, or a weak, unfit host, which is an easier target. For example, louse flies can take larger blood meals from chicks in good body condition, but face weaker immune defences from chicks in poor body condition (Bize *et al*., 2008). Similarly for a predator, dragonflies gain more resources by catching larger prey individuals, but these can fly more quickly and are therefore more difficult to catch (Combes *et al*., 2013). Such a trade‐off between quality and vulnerability has been described in disparate systems (e.g. Carroll & Cramer, 1985; Henaut, 2000; Tschirren *et al*., 2007; Bize *et al*., 2008; Rueesch, Lemoine & Richner, 2012; Cornet *et al*., 2014; Václav & Valera, 2018; Johnson *et al*., 2019), but has not previously been generalised across different classes of antagonists.

Here, I bring together studies on a range of antagonists, including pathogens, host‐consuming parasites, parasitic genetic elements, klepto‐parasites, brood parasites, and predators (including herbivores) (Courchamp, Grenfell & Clutton‐Brock, 2000; Pollock *et al*., 2021), to illustrate that this trade‐off is fundamental. The principles apply to both parasite–host and predator–prey interactions because these function analogously in many ways: both involve the antagonist taking resources from the host or prey (Combes, 2001; Raffel, Martin & Rohr, 2008). Differences between parasites and predators may affect the relative importance of victim quality and vulnerability (see Section III), but not the applicability of the trade‐off itself. For simplicity, I mainly use the terms “host” and “parasite”. Throughout, such statements also apply to predators and prey.

When asking how parasites choose their hosts, “choice” refers to two distinct processes. First, parasites can make decisions (choices) in ecological time; for instance, behavioural plasticity may allow a mosquito to target one host individual over another to feed on (Wynne, Lorenzo & Vinauger, 2020). Second, parasites can evolve to specialise on particular host species (Poulin, 2007), which is not a plastic decision but a hardwired decision selected in evolutionary time. For instance, a brood‐parasitic cuckoo might evolve to parasitise one potential host rather than another (Medina & Langmore, 2016; Langmore *et al*., 2024). An evolutionary “choice” may develop from a behavioural preference (Kawecki, 1998; Vorburger, 2022). I include both processes as choices affected by host quality and vulnerability.

I define host quality from a parasite's perspective as the value of resources potentially available from a host. I define host vulnerability as features of a host that determine how easily a parasite can access its resources (with low vulnerability including harm that host defences could inflict on a parasite). These definitions apply across levels of biological organisation, from intra‐ or inter‐cellular parasites (e.g. Devil Facial Tumour Disease) to colony‐level parasites (e.g. socially parasitic ants) (Foitzik *et al*., 2001; McCallum, 2008; Dixit, 2024).

In this review, I first identify scenarios where a trade‐off between quality and vulnerability will apply. These require mechanisms that create a correlation between those two traits in hosts, and for parasites to make an informed choice. In some cases, the trade‐off will not affect host choice, and host use will instead be explained by constraints, such as those imposed by host availability, competition between parasites, or sensory systems (Mideo, 2009; Thiemann *et al*., 2011; Johnson *et al*., 2019).

Having outlined when a quality–vulnerability trade‐off is likely to apply, I suggest factors that could allow us to predict whether quality or vulnerability will be more important in a given system. I then explore the implications of adopting this framework for our understanding of antagonistic interactions. These include resolving contradictory findings across different studies, examining the effect of global change on host–parasite dynamics, and new perspectives on coevolutionary adaptations. I conclude with suggestions for future research directions.

## II. WHEN DO PARASITES FACE A TRADE‐OFF BETWEEN HOST QUALITY AND VULNERABILITY?

A trade‐off between host quality and vulnerability (*i*) requires a negative correlation between these two factors, and (*ii*) will only affect host choice when a parasite both is able to, and benefits from, differentiating between hosts. How prevalent are situations in which these requirements are met?

### (1) When there is a negative correlation between host quality and host vulnerability

**Fig. 1:**
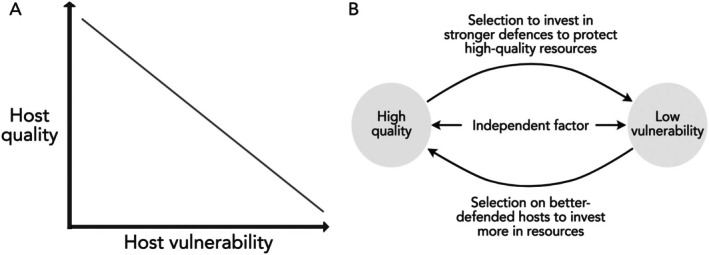
(A) A negative correlation between host quality and vulnerability, where quality is the value of resources a host has, and vulnerability is the ease with which a parasite can obtain those resources. (B) Three types of mechanism that could give rise to this correlation. There are two direct mechanisms, and one in which an independent factor connects quality to vulnerability. Examples of independent factors that could link quality and vulnerability are given in Fig. 2.

**Fig. 2:**
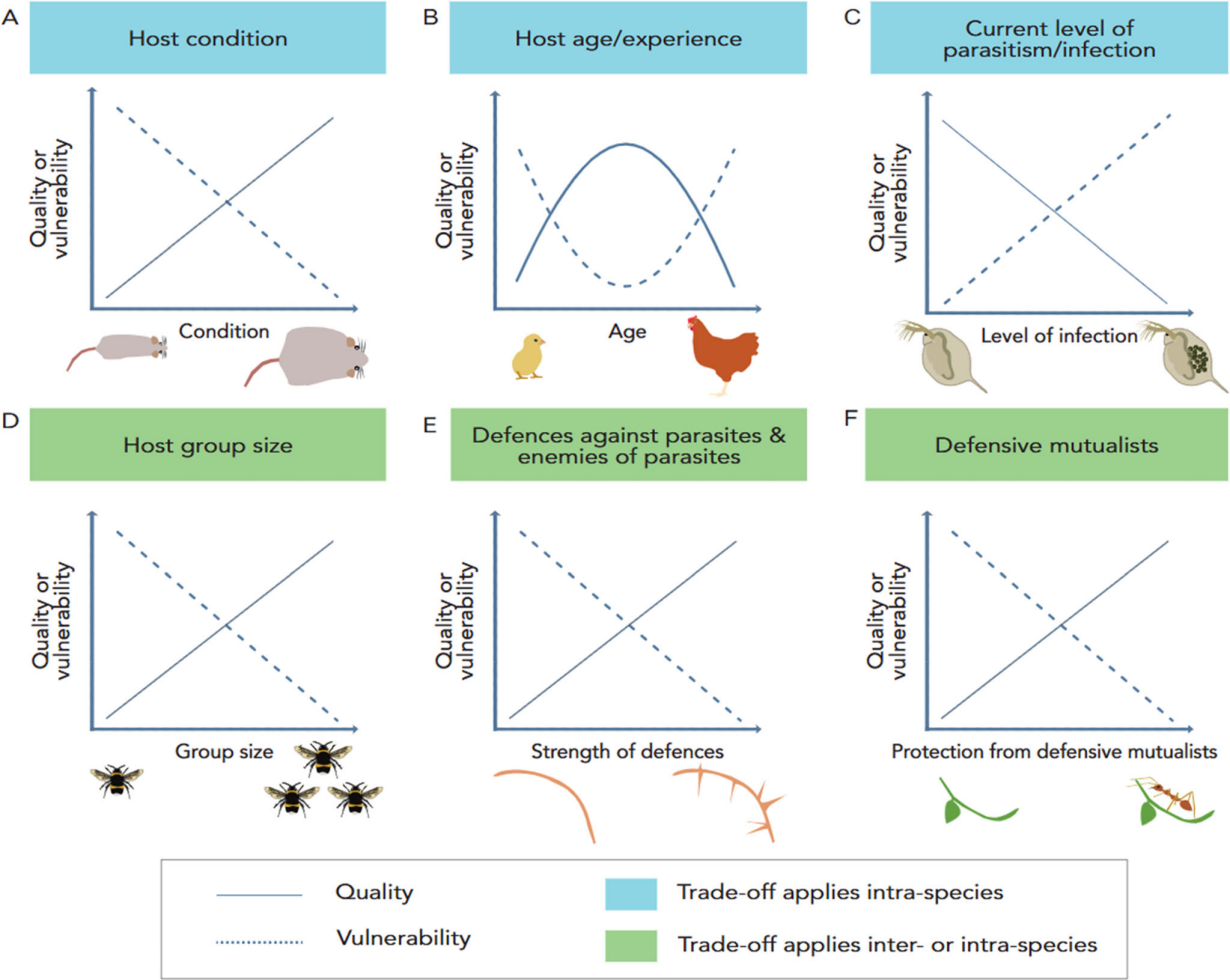
A negative correlation between quality and vulnerability will result when some factor influences quality and vulnerability in opposite ways. For example, in A, host individuals in better condition will be of increased quality (i.e. have more resources available) for the parasite, but will also be of lower vulnerability, because they have stronger defences against parasites. See main text for explanation of factors B–F. The exact shape of the relationship between quality and vulnerability varies, and will affect how a parasite resolves the trade‐off.

A trade‐off is only introduced when increasing quality is concomitant with decreasing vulnerability, because then parasites cannot simply choose hosts that are both highly vulnerable and high quality (Fig. 1A). A negative correlation can arise in three ways (Fig. 1B). Quality and vulnerability can create selection pressures on each other: hosts with high‐quality resources might be under selection to invest in stronger defences to protect those resources; or hosts might only benefit from evolving a life‐history strategy with investment in resources that antagonists want (such as eggs, nectar or food stores) if they are well defended. Additionally, various independent factors can create a negative causal link between quality and vulnerability in hosts. These factors are listed in Fig. 2 and discussed in subsections II.1.*a*–*f* below. They are neither mutually exclusive nor exhaustive, but serve as a starting point to identify situations in which a trade‐off could apply.

#### (a) Host condition

Hosts in good condition might have more resources available for a parasite to exploit, but often also have stronger defences (i.e. lower vulnerability) (Rueesch *et al*., 2012; Garrido‐Bautista *et al*., 2022) (Fig. 2A). Direct evidence for this trade‐off has been established experimentally using food supplementation or restriction to manipulate hosts' body condition (Pulkkinen & Ebert, 2004; Bize *et al*., 2008). For example, *Xenopsylla ramesis* fleas experience fitness effects from the body condition of their rodent host *Meriones crassus*. Flea egg production was significantly higher when parasitising underfed hosts compared to control hosts, likely because underfed hosts were immunosuppressed and so more vulnerable. However, these hosts were also of lower quality, which adversely affected the development time of flea and egg larvae (Krasnov *et al*., 2005).

Condition can affect both the vulnerability and quality of hosts targeted by pathogens too. With better nutrition and therefore condition, hosts can invest more resources in immunity, resulting in reduced vulnerability and therefore lower pathogen virulence (Pike, Lythgoe & King, 2019). On the other hand, better nutrition also improves host quality, and so pathogens can benefit from accessing more resources, leading to increased virulence (Pike *et al*., 2019). A meta‐analysis of host nutrition on pathogen virulence found no overall effect, but did observe high heterogeneity between studies. In some systems the first mechanism was dominant (i.e. reduced vulnerability had the greatest effect), whereas in other systems the latter mechanism was dominant (i.e. improved quality had the greatest effect) (Pike *et al*., 2019).

#### (b) Host age or experience

Increasing competence with age can causally link both quality and vulnerability in hosts (Forslund & Pärt, 1995; Ridley & Child, 2009; Wunderlich *et al*., 2022). This applies to hosts of kleptoparasites, as older, more experienced individuals develop both better foraging abilities and behavioural defences for evasion (Carroll & Cramer, 1985; Tershy, Breese & Meyer, 1990). Parasites can either target juveniles for an easy source of low‐value food items (“like taking candy from a baby”); or attempt to overcome adult defences in order to obtain higher‐value items (“like taking caviar from a middle‐aged man”). Behavioural defences and quality can improve with age as a function of learned experience, or be linked to physical maturation [as with skin thickness forming a barrier to mosquito bites or helminth infection (Edman & Scott, 1987; Lewert & Mandlowitz, 1963)].

A host's age can also have non‐linear effects on vulnerability to parasitic diseases; this applies across a range of vertebrate and invertebrate hosts for infections by fungi, bacteria, viruses, trematodes and microsporidians (Müller, Fülöp & Pawelec, 2013; Ben‐Ami, 2019). Underlying mechanisms include immune priming and senescence, which can drive the effect of age on vulnerability in opposite directions. The relationship between vulnerability and age then changes over time, initially decreasing before increasing (Fig. 2B). Simultaneously, a host's age can affect its *quality* with respect to parasite transmission – particularly if parasite‐induced mortality occurs more rapidly in very old or young hosts, limiting the window for parasite transmission (e.g. Gipson & Hall, 2018). We can infer that a trade‐off could then result, when the effect of age on quality is the inverse of its effect on vulnerability.

#### (c) Current level of parasitism/infection

Hosts that are already infected may be more vulnerable to further parasitism (by the same or another species) as the original parasites weaken host defences (Vicenzi *et al*., 2007; Grogan *et al*., 2018). This occurs directly when parasites induce immunosuppression, such as with HIV (Vicenzi *et al*., 2007), or the chytrid fungus in amphibians (Grogan *et al*., 2018); or occurs indirectly as parasites' consumption of host resources leads to weakened defences (Koski & Scott, 2001; Cornet & Sorci, 2010). Either mechanism creates a positive feedback loop between parasitism and vulnerability (Beldomenico & Begon, 2010). However, competition between parasites for host resources may mean that an already‐infected host is low quality from the perspective of a prospective parasite (Vannatta *et al*., 2020). For example, within‐host competition for resources reduced the success of microparasites infecting *Daphnia magna* (measured as the number of spores that grew and matured) (Ebert, Zschokke‐Rohringer & Carius, 2000). There is thus evidence from some systems that already‐parasitised hosts have lower quality, and from others that they have higher vulnerability (Woolhouse *et al*., 2002). If these effects are combined in the same system, the result is a trade‐off between host quality and vulnerability (Fig. 2C).

This trade‐off also results when trophically transmitted parasites increase their host's vulnerability to predation. This occurs *via* manipulation of the intermediate host; for example, gammarids infected by acanthocephalan parasites become attracted to, rather than avoiding, their fish predators (the final host of the parasite) (Perrot‐Minnot, Kaldonski & Cézilly, 2007). To a predator, the quality of infected and uninfected prey may initially be similar: the same quantity of resources is available. However, predators incur costs later when the parasite establishes itself in them as a final host. This makes infected hosts lower quality to the predators, while also more vulnerable due to the behavioural manipulation.

Depending on the specifics of a system, prior parasitism can create the same trade‐off in the opposite way: increasing quality and decreasing vulnerability. In *Daphnia magna*, individuals infected by iridovirus DIV‐1 have higher energy content compared to uninfected individuals, but the previous infection incurs longer handling times for their *Notonecta* predators – making them higher quality, but less vulnerable (Prosnier *et al*., 2024). Prior infection could also lead to lower vulnerability in already‐parasitised hosts due to cross‐immunity (Bhattacharyya *et al*., 2015).

#### (d) Host group size

Quality and vulnerability can become correlated when multiple hosts are targeted collectively rather than as individuals (e.g. eusocial colonies or groups of co‐operative breeders). Larger group sizes will tend to be higher quality overall but less vulnerable to attack (Fig. 2D). For brood‐parasitic cuckoo bumblebees [*Bombus* (subgen. *Psithyrus*) spp.], selecting a host colony with many workers secures effective care for their offspring, but comes with an increased risk of being repulsed or killed while infiltrating the nest (Sramkova & Ayasse, 2009; Lhomme & Hines, 2019). By contrast, parasitising a colony with few workers is easier for the cuckoo bumblebee, but means fewer workers are then available to rear their brood. The optimal strategy for *Bombus vestalis* appears to be selecting colonies of around 10–15 workers, an intermediate between five workers (in which all parasitic females survived) and 20 workers (in which 30% of parasitic females were killed) (Sramkova & Ayasse, 2009).

The principle of host group size affecting quality and vulnerability extends across taxa, including to socially parasitic ants (Pohl & Foitzik, 2011) and avian brood parasites of co‐operatively breeding hosts. Larger breeding groups are harder to parasitise, but offspring of successful parasites subsequently experience better care (Poiani & Elgar, 1994; Canestrari, Marcos & Baglione, 2009; Feeney *et al*., 2013). Similarly, larger groups can have increased foraging success but also stronger repulsion of kleptoparasites, as observed in wild dogs *Lycaon pictus*. Larger hunting groups have higher capture success but can defend kills more effectively against kleptoparasitic spotted hyenas *Crocuta crocuta* (Carbone, du Toit & Gordon, 1997).

#### (e) Defences against parasites and enemies of parasites

In this scenario, front‐line defences reduce a host's vulnerability through preventing egg‐laying by brood parasites, but also increase a host's quality if they function similarly to defend nests (and therefore parasitic offspring) from predators (Fig. 2E). For example, aggression by host birds acts as a frontline defence against brood parasites, while also functioning against nest predators, thereby benefitting a parasite chick in a host nest (Attwood *et al*., 2023). Other front‐line defences, such as parental attentiveness or nest placement and structure, could have a similar effect (Brown & Brown, 1991; Feeney, Welbergen & Langmore, 2012). Behaviours that function in this way can also correlate with age and/or experience (see Section II.1.*b*). Horsfield's bronze cuckoos *Chalcites basalis* choose to parasitise younger (less‐experienced) female superb fairy‐wrens *Malurus cyaneus*, possibly because these individuals have less effective behavioural defences against the cuckoos (Langmore & Kilner, 2007).

#### (f) Defensive mutualists

In defensive mutualisms, a species protects its host from natural enemies (Hopkins, Wojdak & Belden, 2017). The existence and strength of these mutualisms by definition directly reduce a host's vulnerability to parasites (Fig. 2F). At the same time, there is evidence that some defensive mutualists select their host based on quality, with higher‐quality hosts attracting more mutualist partners (e.g. *Vochysia elliptica* plant hosts attracting defensive ant mutualists; Pacelhe *et al*., 2019). When the features that define host quality to a defensive mutualist are the same as (or correlated with) those for a parasite, a causal link between host quality and vulnerability becomes established. This may occur with extrafloral nectar production by some plants, which attracts ants that defend the plants from nectar robbers (Irwin *et al*., 2010).

#### (g) The trade‐off at different scales

**Fig. 3:**
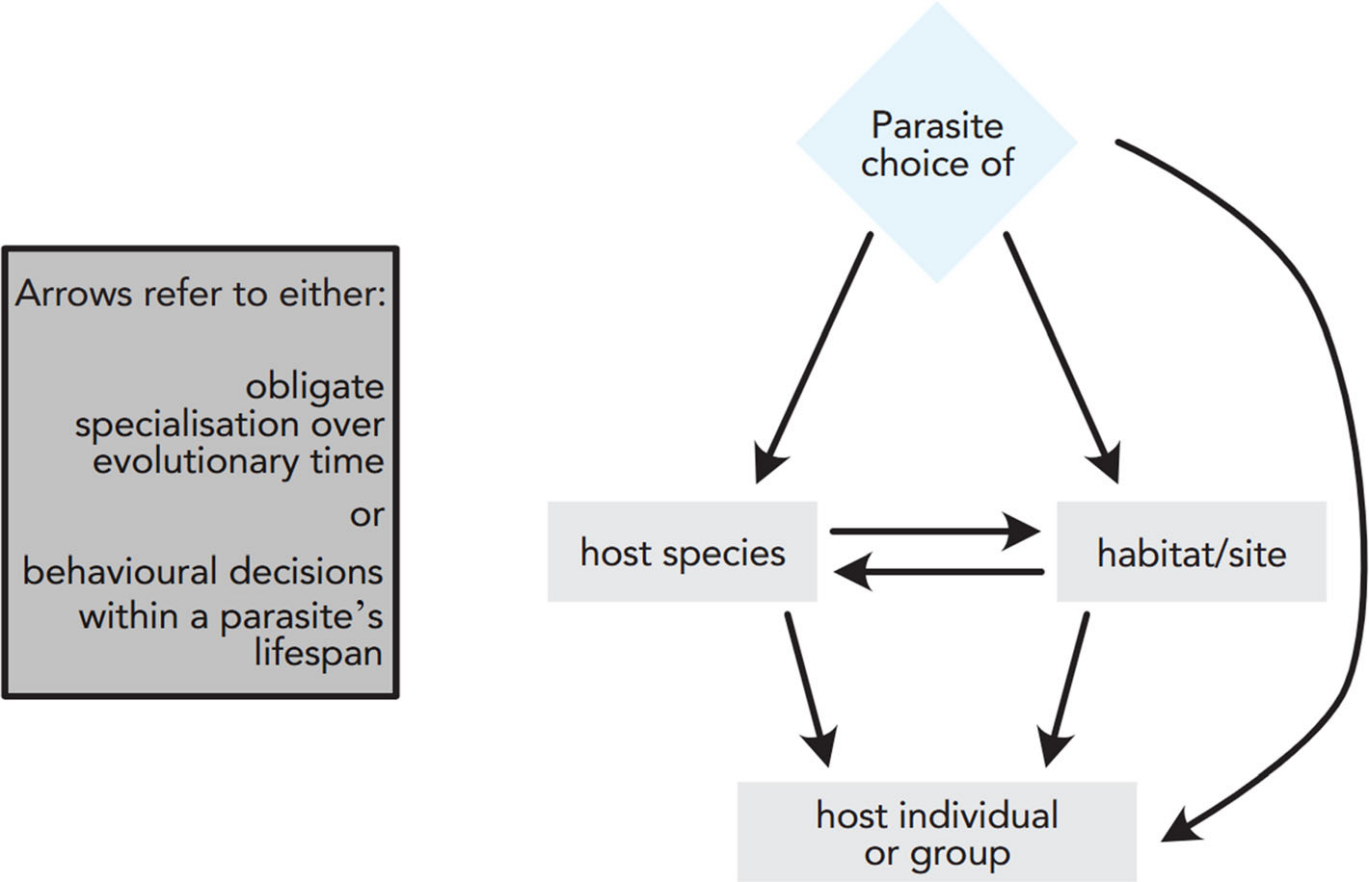
A decision tree of nested choices parasites may face. Parasites (highlighted in blue) may face a trade‐off between quality and vulnerability in different habitats; having selected a habitat, this may then determine the range of host species available (another choice potentially involving a quality–vulnerability trade‐off); and the choice of host species may then affect the range of quality and vulnerability across individual hosts. The trade‐off can therefore exist at multiple spatial and temporal scales. At each scale, the decision could occur as evolutionary specialisation or as individual plasticity.

The quality–vulnerability trade‐off can occur at multiple spatial and temporal scales, with the options available at each scale dictated by prior choices (Fig. 3). For instance, a brood‐parasitic cuckoo faces a choice between hosts of differing quality and vulnerability within their territory, but the number and range of hosts that are available will depend on their choice of breeding site (Langmore & Kilner, 2007). Selecting a breeding site is a coarser‐scale decision which can also involve a trade‐off between quality and vulnerability. Areas of high host nest densities have great potential for exploitation (i.e. may be considered high quality), but may also be of low vulnerability due to higher overall host vigilance and/or social learning. Similar landscape‐scale trade‐offs occur for predators deciding where to forage. Little penguins *Eudyptula minor* choose to forage in patches where low‐quality prey (including larval fish, anchovy and sprat) can be captured at high rates. This is suggestive of a trade‐off resolved in favour of vulnerability (capture effort) over quality (calorific gain) (Sutton & Arnould, 2022). Having selected a foraging patch, the penguins may then face another choice between prey groups/individuals, with another trade‐off between quality and vulnerability. Decisions relating to victim choice may therefore be nested within each other, constraining the range of victims that antagonists can subsequently choose between (Fig. 3).

#### (h) When a negative trade‐off is not expected

It is also important to consider when a negative correlation is *not* expected. For instance, host mobility or exploratory behaviour could increase both vulnerability *via* exposure to parasites, but also host quality in terms of future transmission opportunities (Teitelbaum *et al*., 2018). Similarly, larger hosts may be more vulnerable to ectoparasites (due to their greater surface area), while also representing a higher‐quality resource (Poulin, 2013). A positive correlation between quality and vulnerability could also arise when investment in parasite defences reduces the energy available for hosts to invest in other aspects of their physiology (Sheldon & Verhulst, 1996, Giolai & Laine, 2024). Whenever a positive correlation exists, the optimal choice for a parasite is straightforward: maximise both quality and vulnerability simultaneously. Where a negative correlation exists, the optimal choice is less obvious, and the trade‐off is expected to resolve differently based on the specifics of a given system.

Nevertheless, the existence of a negative correlation between host quality and vulnerability alone is not sufficient for the trade‐off to affect a parasite's choice. It must also be possible for parasites to adopt their optimal strategy, which I consider next.

### (2) When the parasite's sensory and cognitive abilities enable it to differentiate between hosts, and the parasite benefits from doing so

**Fig. 4:**
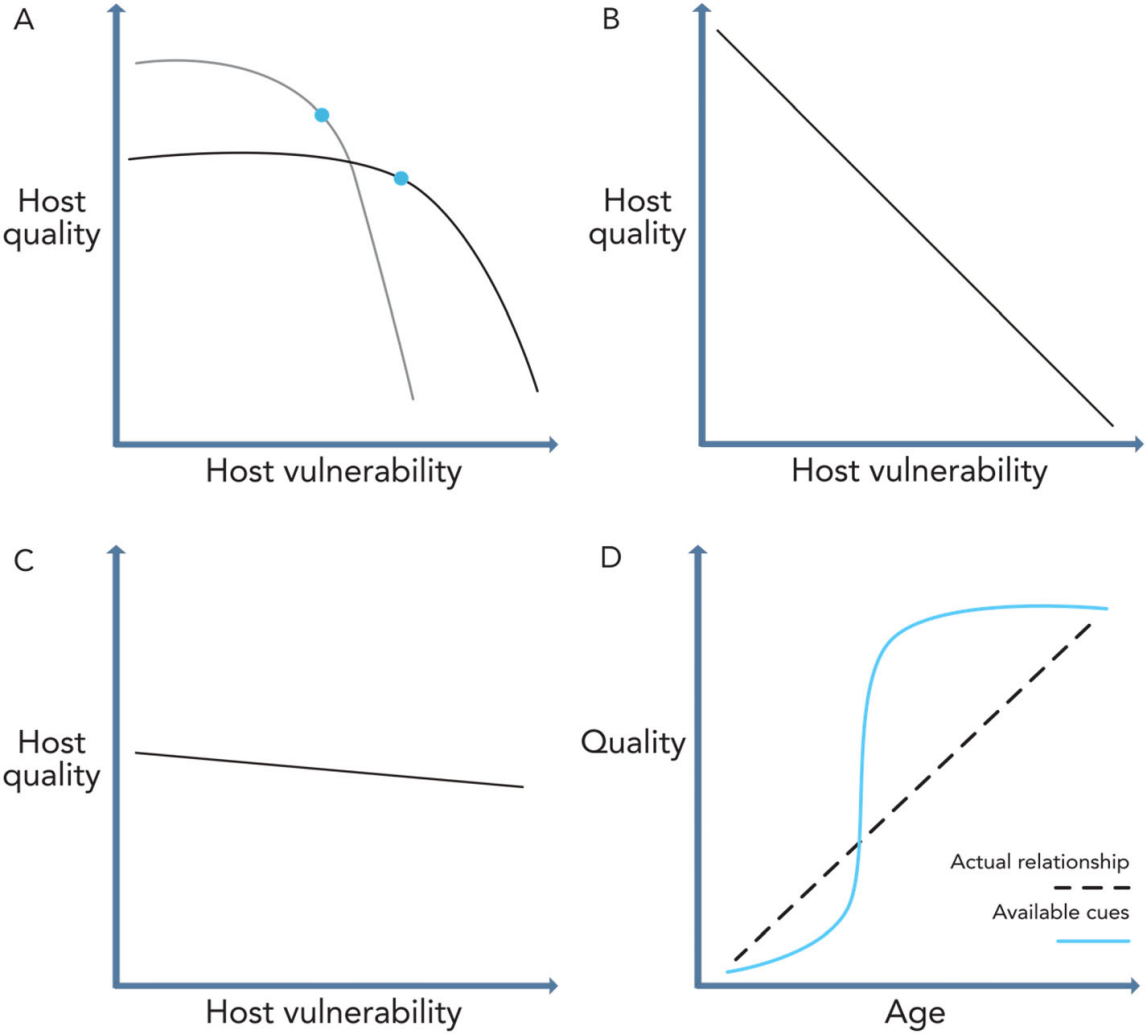
(A) A trade‐off between host quality and vulnerability will exert effects on parasite choice when there are clear solutions providing higher fitness (blue dots). Both quality and vulnerability are plotted in units of fitness that these represent to the parasite. Discrepancies between studies on parasite choice might arise when the shape of the trade‐off between quality and vulnerability differs between populations of hosts, leading to different optima for parasites. (B) By contrast, all solutions to the trade‐off could provide the same fitness, with the benefits of higher quality exactly set off by the costs of lower vulnerability. There will be no evolutionary response in host choice by parasites in this scenario, or if fitness differences in solutions are smaller than the effects of drift. (C) If the relationship approaches vertical or horizontal, this implies that one trait matters much more to parasite fitness (i.e. parasites would base their choice mainly on host vulnerability in this example). (D) The cues available from hosts may not correlate perfectly with their true quality or vulnerability. For example, pelican plumage changes take place stepwise with age, while foraging quality may increase linearly. In these instances, parasite choice may diverge from the optimum as decisions can only be based on available cues.

Even where a negative correlation between host quality and vulnerability exists, it is only relevant to parasite choice if there are clear solutions to the trade‐off (Fig. 4A–C), and if the parasite is capable of detecting and discriminating relevant variation among hosts (Fig. 4D). Cues of host quality or vulnerability may occur in a range of modalities, including olfactory, auditory and visual. Hosts may actively signal their low vulnerability to parasites (or prey may signal this to predators, e.g. stotting in Thomson's gazelles *Gazella thomsoni*; FitzGibbon & Fanshawe, 1988). Alternatively, hosts might signal their quality to potential mates, presenting parasites with an opportunity to eavesdrop. However, the cues needed for parasites to choose between hosts may not always be available (Langmore & Kilner, 2007; Cozzarolo *et al*., 2019).

This has the potential to explain seeming deviations from predicted optima. For intra‐specific kleptoparasitism of food items by Carib grackles (*Quiscalus lugubris*), individuals that immerse their food in water (“dunking”) create higher‐quality resources as this food can be ingested more easily. Yet kleptoparasitic grackles did not attempt to steal dunked items more often than dry ones. The authors suggested that the lack of preference was because there was no obvious visual cue grackles could use to distinguish between dry and dunked items (Morand‐Ferron, Veillette & Lefebvre, 2006). By contrast, kleptoparasitic gulls and terns were found to target pelicans that had foraged successfully (i.e. were higher quality) (Shealer, Floyd & Burger, 1997). Unlike in the Carib grackle system, a clear visual cue exists to distinguish higher‐quality pelicans, in the form of bill submersion time. Successful birds submerge their bills for longer, to drain water out, while unsuccessful birds immediately expel water and thus have their bills submerged for a shorter time. The presence of such cues is critical for enabling parasites to make an informed choice.

In other cases, cues may be present but correlate imperfectly with quality and/or vulnerability. Plumages of brown pelicans (*Pelecanus occidentalis*) differ between adult and juvenile birds, and kleptoparasites can use this visual cue to distinguish between birds of different ages. However, the plumage changes are stepwise and occur relatively early in the pelicans' lifespan, with fewer cues beyond the definitive plumage at 3–5 years of age (Shields, 2020). Therefore, even if quality and/or vulnerability changes linearly with pelican age, we may expect kleptoparasites to discriminate only between pelicans with plumage differences (Fig. 4D). Discriminating between a 5‐year‐old pelican and a 7‐year‐old pelican is probably more challenging, because these individuals are inseparable based on plumage.

Beyond being imperfect, host cues can actively mislead, such as when parasites manipulate hosts into being more attractive to vectors or predators (Moore, 2002; Poulin, 2010). For example, a nematode parasite causes *Cephalotes atratus* ants to display conspicuous red gasters, thought to resemble berries and make them more attractive to frugivorous birds [the definitive hosts for the nematode (Yanoviak *et al*., 2008)]. The parasite causes a cue of quality to change without increasing the actual resource quality. Similarly, infections by a range of parasites can make hosts more attractive to arthropod vectors than non‐infected hosts, although sometimes this pattern is absent or even reversed (reviewed in Cozzarolo *et al*., 2020). Discrepancies between these studies might be partly explained by a parasite's effect on both quality and vulnerability.

Even when accurate cues are available, detecting these depends on the sensory and cognitive systems of the parasite. Limited sensation or cognition may explain the lack of graded host selection by some parasites and predators (Janz & Nylin, 1997). For example, big‐eyed bugs *Geocoris punctipes* select nutritionally inferior prey (pea aphids *Acyrthosiphum pisum*) over eggs of the corn earworm *Helicoverpa zea*. This appears to be because big‐eyed bugs detect prey visually from movement, and have limited ability to detect sessile prey (Eubanks & Denno, 2000). Such sensory or cognitive constraints can channel the evolution of parasite choice.

However, these constraints may not apply uniformly to a parasitic population. Older parasites could develop improved cognition or have more opportunities for individual and social learning. This would reduce constraints as their discriminatory abilities improve over time. This could explain the finding that adult gulls targeted juvenile pelicans for kleptoparasitism, while first‐winter gulls selected pelicans at random (Carroll & Cramer, 1985). Natural selection also has the potential to remove sensory or cognitive limitations to parasite choice, through selecting for detection capabilities. The ability of oncomiracidia ectoparasites to use glycoproteins as chemosensory cues to target particular host fish may be one such example (Wunderlich *et al*., 2022). In general, parasites that actively seek their victims using cues are more likely to choose hosts based on quality and vulnerability than those that encounter hosts passively (Trillo, Bernal & Hall, 2023).

As well as the *ability* to differentiate between hosts, a parasite must *benefit* from doing so in order for the quality–vulnerability trade‐off to affect host choice. Sometimes a parasite's fitness is best served by saturating all available hosts, rather than by being choosy; this can be due to the time and energetic costs of parasitism, including the availability of hosts (a bad host often being better than no host) (Manzoli *et al*., 2021).

If the fitness benefits from selecting particular hosts are relatively small, then multiple types of cost could prevent parasite choosiness. For parasites with a short window of opportunity for attack, the time it takes to discriminate between hosts is especially costly. Competition among parasites – such as territoriality in brood‐parasitic cuckoos – could also limit parasite choice to the degree that it becomes evolutionarily irrelevant. Separately, there may be energetic costs of using sensory/cognitive systems for discrimination, or costs of movement to and between hosts. This appears to apply to ticks (*Ixodes ricinus*), as their low movement speeds and inability to jump or fly likely increases the costs of attempting to switch hosts. Where other, more mobile, ectoparasites preferentially infested avian chicks of particular seniorities or ages, ticks were found to infect chicks at random (Roulin *et al*., 2003).

In this section, I have highlighted two factors that prevent free parasite choice of host: sensory/cognitive inability to discriminate between hosts, or insufficient benefits accruing from such discrimination. Some studies use the absence of host preferences to imply that just one of these factors applies – such as non‐specific host preference by mosquitoes suggesting that “blood source and quality are irrelevant for their reproductive fitness” (Takken & Verhulst, 2013, p. 434). However, it remains possible that blood quality is important for mosquito fitness, and that instead, limitations of their sensory/cognitive systems explain the lack of preference. Ideally, future research will consider both options, or even use empirical tests to distinguish between alternative explanations (Section V.1).

When the costs of discriminating between hosts are sufficiently small, the cues to do so are available, and there is a negative correlation between host quality and vulnerability with clear solutions to this trade‐off, then these solutions are expected to affect host preferences. Given that this appears to be the case within multiple types of host–parasite interactions, can we then predict whether a parasite will choose their host primarily based on quality or vulnerability?

## III. PREDICTING WHICH OF QUALITY OR VULNERABILITY WILL DETERMINE A PARASITE'S CHOICE

The optimal host for a parasite will depend on the particular relationship between quality and vulnerability in a given system (Fig. 4A). Once this relationship is established, we can use optimality modelling (well‐established in behavioural ecology; Davies, Krebs & West, 2012) to predict parasite choice. Here, I suggest there are specific features of host and parasite ecology that can be used *a priori* to predict which of quality or vulnerability will have the greater effect on parasite choice (Table 1).

**Table 1:** Factors that could predict when parasites will favour high‐quality *versus* highly vulnerable hosts.

When quality has a greater effect on parasite choice	When vulnerability has a greater effect on parasite choice
The resource requirements of the parasite represent a significant proportion of host resources	The resource requirements of the parasite represent a small proportion of host resources
There is minimal variation in vulnerability across hosts	There is minimal variation in quality across hosts
Some hosts are of such low quality that they cannot support the parasite at all	Some hosts have defences which entirely prevent infection/attack
A parasite has evolved adaptations that increase a host's vulnerability	A parasite can induce changes in the host's quality itself (e.g. parasitic castration)
Host activity/effort is important to parasite transmission (e.g. brood parasitism where successfully raising the parasite chick is “transmission”)	Parasite transmission can occur passively (e.g. between vertebrate hosts of vector‐borne diseases)
A parasite targets just one or very few hosts over its lifetime	A parasite targets many hosts over its lifetime

### (1) The proportion of host resources a parasite needs

The relative resources of the host and parasite affect the importance of host quality. Quality is particularly important when the parasite's resource requirements represent a substantial proportion of host reserves (Ebert *et al*., 2004). When a parasite takes a small amount of resources relative to the host's total reserves, quality may matter less because effective variation in quality between hosts is reduced. In the latter case, parasite growth and reproduction may not be much limited by between‐host differences in resources, and we would instead expect vulnerability to play the main role in determining parasite choice.

This fits with the pattern that most evidence of quality superseding vulnerability comes from parasite–invertebrate host systems (Ebert *et al*., 2000, 2004; Pulkkinen & Ebert, 2004; Bedhomme *et al*., 2004; Seppälä *et al*., 2008), while most evidence of vulnerability superseding quality is from parasite–vertebrate host systems (Koski & Scott, 2001; Krasnov *et al*., 2005). This is expected if parasites consume a higher proportion of their host's resources in invertebrates (to which they are often more similarly sized) compared to vertebrates (Seppälä *et al*., 2008). A meta‐analysis looking at the effect of host nutrition on parasite virulence found evidence of this pattern. Parasites showed a decrease in fitness in resource‐limited invertebrate hosts (due to fewer host‐derived resources for the parasite), but an increase in fitness in resource‐limited vertebrate hosts (due to weaker host immunity and therefore increased vulnerability) (Pike *et al*., 2019).

### (2) Variation in either quality or vulnerability is minimal

If variation in either trait (quality or vulnerability) is relatively small, there may be no selection for a parasite to differentiate based on that trait (Fig. 4C). Little variation in vulnerability across hosts would lead us to expect quality to play the deciding role, and *vice versa*.

*Lymnaea stagnalis* snails experiencing starvation show very little change in their vulnerability to trematode parasites (*Diplostomum spathaceum*), but have fewer resources for the parasites to exploit: quality (rather than vulnerability) determines parasite success in this system (Seppälä *et al*., 2008). Similarly, ladybirds *Adalia bipunctata* lack an effective immune response to mites (*Coccipolipus hippodamiae*), so starvation affects resource availability without impacting vulnerability. Mites have reduced fecundity on starved ladybirds, demonstrating that quality is more important in this parasite–host pair as well (Ryder, Hathway & Knell, 2007). Quality will inevitably play a greater role when variation in vulnerability is minimal.

The importance of variation is also seen in studies on kleptoparasites of pelicans. When pelican quality varied widely across age brackets – with adult pelicans more than twice as successful at foraging than immatures – adult pelicans were attacked more often (Tershy *et al*., 1990). This implies that quality drove the kleptoparasitic gulls' choice. This contrasts with a different study population in which pelican quality showed little variation across age brackets, and adults were only 9% more successful than immatures (Carroll & Cramer, 1985). Vulnerability was more important to parasite choice in this study, which found that *juvenile* pelicans were the preferred victims of kleptoparasitic gulls.

As well as quality variation, differences in vulnerability variation can explain contrasting findings for kleptoparasite host choice. Kleptoparasitic fork‐tailed drongos *Dicrurus adsimilis* targeted (low‐quality) juvenile pied babblers *Turdoides bicolor* (Ridley & Child, 2009). The authors compared their findings to a study in which kleptoparasitic gulls and terns targeted (high‐quality) adult brown pelicans (Shealer *et al*., 1997). They suggested that a key difference between the two systems was variation in hosts' handling time of food items. Pied babblers showed distinct differences in handling time (a feature of vulnerability) with age, whereas brown pelicans in this study showed no such variation. The minimal variation in vulnerability for pelicans meant parasites selected hosts on the basis of quality, whereas parasites of babblers made choices based on vulnerability.

Effects of host quality on parasite fitness may also be non‐linear, such that above a certain level further variation has minimal effect on parasite fitness. Microsporidian parasite spore production was positively influenced by food availability for their mosquito hosts, but this effect became weaker at higher food levels (Bedhomme *et al*., 2004). Quality may therefore initially have the deciding role for host choice, but becomes saturating at high food levels. We would then expect parasites to switch to selecting between well‐fed hosts on the basis of their vulnerability.

### (3) A parasite cannot survive/reproduce on certain hosts due to either factor

In the extreme, there may be a binary choice between hosts on a which a parasite can reproduce, and those on which it cannot (either due to the hosts' quality or vulnerability). In such cases, this binary choice necessarily overrides considerations of the other factor. This is the case for many solitary insect parasitoids, for which only one parasitoid egg can develop per host (Ortiz‐Martínez *et al*., 2019; Chen *et al*., 2020). Even though already‐parasitised hosts may be more vulnerable, to a parasitoid they are of such low quality that they should always be rejected.

This is also illustrated by parasitic genetic elements that replicate to the detriment of the genome [e.g. transposons, segregation distorters and B chromosomes (Camacho, Sharbel & Beukeboom, 2000; Robillard *et al*., 2016)]. Because genes in somatic cells are not passed on to future generations, the genome in somatic cells is of zero quality to parasitic genetic elements. We would therefore expect these elements preferentially to parasitise the genome in germline cells (Johnson, 2008; Bourque *et al*., 2018). In line with this, there is evidence that transposons evolve to be silent in somatic and active in germline cells (Siebel & Rio, 1990; Fischer, Wienholds & Plasterk, 2001), despite germline cells having lower vulnerability than somatic cells (Haig, 2016). This lower vulnerability is due to evolved defences that restrict the activity of transposons, such as delayed zygotic genome activation (Haig, 2016). Nevertheless, the lower vulnerability is irrelevant to transposon fitness, because only genes in the germline are passed on, and therefore of sufficient quality.

### (4) A parasite has adaptations that can manipulate host traits

Parasites can evolve adaptations to alter a host's quality or vulnerability (McCarthy, Fitzpatrick & Irwin, 2004; Kuroda *et al*., 2024). This minimises the effective variation in that trait between hosts, so host choice can be made on the basis of the other trait. Some flea species have combs (ctenidia), which help them to anchor and resist grooming by their desert rodent hosts. This adaptation means that these species do not experience differences in host vulnerability, and could explain why two such species do not distinguish between hosts, unlike three other species which lack ctenidia (Krasnov, Khokhlova & Shenbrot, 2003). Any parasitic adaptations that overcome host defences effectively manipulate host vulnerability.

Host quality could be manipulated *via* parasitic castration. This is where parasites induce hosts to divert resources from reproduction to growth, such that hosts cease to reproduce (Baudoin, 1975; Lafferty & Kuris, 2009). If parasites can increase host quality post‐infection, their choice of host is more likely to be based on vulnerability instead. Hypothetically, this could also occur for brood parasites taking advantage of ineffective nest defence by poor‐quality hosts. Chicks could subsequently manipulate their foster parents into being high‐quality parents, by inducing higher‐than‐usual feeding rates through brood mimicry or supernormal stimuli (Spottiswoode, Kilner & Davies, 2012). Hairworms also manipulate the quality of the sexually cannibalistic mantids they parasitise. Parasitised males were less likely to engage in courtship and mounting behaviour than non‐parasitised males, increasing the likely length of time they could be exploited as a host (Kuroda *et al*., 2024).

### (5) Mode of parasite transmission

The more host effort required for transmission, the more important “host quality” will be. For example, sexually transmitted diseases require hosts to be sexually active to transmit them, more so than vector‐borne diseases. Host quality is therefore more important for parasites that are sexually transmitted (Ryder *et al*., 2007) or require high energy inputs for transmission (e.g. most brood parasites, or vectors themselves as intermediate hosts). By contrast, host quality matters less for parasites that can be passively transmitted.

### (6) The number of hosts a parasite targets over its lifetime

If a parasite infects just one host over its lifetime, the quality of that host may be more important than for a parasite that can infect multiple hosts. This is because quality has an ongoing effect on fitness, while vulnerability can be ongoing (multiply infecting parasites) or reduced to a single short event (singly infecting parasites). In singly infecting parasites the fitness effects of quality and vulnerability have different leverage, because quality effects act multiplicatively, while vulnerability effects do not. In multiply infecting parasites both effects act multiplicatively. Choosing hosts of low vulnerability may take priority for parasites infecting multiple hosts because host defences must be overcome during each parasitism event.

### (7) Consequences of predicting relative importance

The relative importance of quality and vulnerability potentially reflect whether the host or parasite is ahead in a coevolutionary arms race. If quality is relatively more important for a parasite's choice, that suggests they are ahead in the arms race. If vulnerability is more important, that suggests hosts are ahead as their defences limit parasite choice. This might lead to oscillations over time in how parasites choose hosts, as each party evolves new counter‐adaptations.

As well as predicting *which* hosts a parasite will target, quality and vulnerability can even predict *whether* it pays a parasite to parasitise. In models of kleptoparasitic behaviour, increasing the length of aggressive contests (i.e. decreasing vulnerability without affecting quality) makes parasitism less common (Broom & Ruxton, 2003). The frequency at which parasitism is beneficial therefore varies based on quality and vulnerability, which could directly affect the behaviour of facultative parasites.

## IV. IMPLICATIONS OF ADOPTING A QUALITY–VULNERABILITY TRADE‐OFF FRAMEWORK

What are the consequences of taking this perspective on antagonistic interactions? I suggest three main impacts of this framework: (*i*) resolving conflicting findings in studies about host/prey choice; (*ii*) applying antagonist choice to project the effects of anthropogenic change on ecosystems; and (*iii*) understanding coevolution and co‐adaptation in antagonistic systems, particularly where hosts/prey alter cues of quality or vulnerability.

### (1) Resolving conflicting findings in studies about host choice

The literature on parasite choice is filled with conflicting findings across a range of different fields. For example, some studies find that parasites choose high‐quality hosts, while others find that parasites prefer low‐quality hosts. These disparities might be explained if hosts differ in vulnerability, creating different optima for the parasite (e.g. Figure 4A). Table 2 pairs studies with contrasting findings, and suggests how a quality–vulnerability trade‐off could explain these discrepancies. I also focus on one of these examples – ectoparasites selecting chicks within nests – in greater detail.

**Table 2:** Examples of contrasting results from similar systems investigating either host quality, host vulnerability, or both.

Studies where highest quality hosts are selected	Studies where highest vulnerability hosts are selected	Common features and how the trade‐off could apply
Gulls preferentially klepto‐parasitised adult pelicans (Tershy *et al*., 1990)	Gulls preferentially klepto‐parasitised juvenile pelicans (Carroll & Cramer, 1985)	Kleptoparasites selecting hosts based on age. A key difference between the studies is the extent to which pelicans differed in quality with age.
Invertebrate predators selected the most nutritious prey (Mayntz *et al*., 2005; Schmidt *et al*., 2012; Williams & Flaxman, 2012)	Invertebrate predators did not select the most nutritious prey (reviewed in Eubanks & Denno, 2000; Schmidt *et al*., 2012)	Insect predators selecting prey based on nutrition (quality). In cases where this was not found, prey vulnerability and/or limitations on the parasite's ability to choose may explain findings.
Bacteria *Pasteuria ramosa* parasites were more successful in well‐fed *Daphnia magna* hosts than in those that were poorly fed (Ebert *et al*., 2000)	Younger *Daphnia magna* hosts were more vulnerable to *Pasteuria ramosa* infection, and early‐infected hosts produced more parasite transmission stages (Izhar & Ben‐Ami, 2015)	Bacterial parasites selecting *Daphnia* hosts based on body condition or age. The shape of the trade‐off between quality and vulnerability may be different for body condition and for age, meaning parasites prefer quality in reference to body condition, but vulnerability in reference to age.
Hen fleas *Ceratophyllus gallinae* laid more eggs on food‐supplemented great tit *Parus major* hosts (Tschirren *et al*., 2007)	*Xenopsylla ramesis* fleas lay more eggs on food‐restricted rodent *Meriones crassus* hosts, although offspring quality was then lower (Krasnov *et al*., 2005)	Fleas selecting hosts based on body condition. The species of flea and host differed between these studies. This may have meant that host quality and vulnerability responded differently to food availability, or that those qualities affect parasite choice differently.
Parasitic flies selected more senior chicks within nests (Dawson & Bortolotti, 1997; Roulin *et al*., 2003; Valera *et al*., 2004)	Parasitic flies selected more junior chicks within nests (Christe *et al*., 1998; Roulin *et al*., 2003; Simon *et al*., 2003)	Parasitic flies selecting chicks within a nest based on age. See Section IV.1.*a* for detailed explanation of how the trade‐off may explain contrasting findings.

#### (a) Case study: the “tasty chick” hypothesis

The “tasty chick” hypothesis posits that ectoparasites will aggregate on the smallest chick within a brood, as this will be the host with the weakest immune system (Christe, Moller & Lope, 1998). It assumes that chick immunocompetence is positively correlated with body condition, in which case the quality–vulnerability trade‐off applies. It also implicitly assumes that parasites will prioritise vulnerability over quality of hosts. This may be a reasonable assumption in the context of a relatively small ectoparasite feeding on a larger vertebrate host. Studies on house martin (*Delichon urbica*) chicks and their hematophagous ectoparasite (the house martin bug *Oeciacus hirundinis*) find evidence in support of the hypothesis: parasites tended to aggregate on chicks which have both a low body condition and weak immune response (Christe *et al*., 1998). Similarly, blow flies (*Protocalliphora spp*.) aggregated on the smallest, lowest‐ranking blue tit (*Cyanistes caeruleus*) chicks in nests (Simon *et al*., 2003), and biting insects preferred to target smaller male nestlings (García‐del Río *et al*., 2024).

However, the picture becomes more complicated across other species and studies. The hematophagous fly *Carnus hemapterus* was found to prefer larger nestlings across a range of species, with one study establishing that this was an active choice (i.e. the trend remained even after accounting for greater habitat availability due to host surface area) (Dawson & Bortolotti, 1997; Valera *et al*., 2004). What creates this divergence in findings? It may be because the basic tenet of a negative correlation between quality and vulnerability is not met in this particular ectoparasite–chick system. Valera *et al*. (2004) suggest that larger chicks could be easier to extract blood from, due to their skin composition or thickness. This would create a positive correlation between quality and vulnerability. Variability in the relationship between quality, age and vulnerability extends to other species: although junior barn owl (*Tyto alba*) chicks have lower humoral immune responses than older chicks, there is no significant difference in the same immune responses for great tit (*Parus major*) chicks according to seniority (Roulin *et al*., 2003). Differences in these host features could well explain the differences observed in parasite choice. Alternatively – or in addition – the balance between quality and vulnerability may vary for different parasite–host pairs. This could explain why some parasite species prefer younger chicks, while older chicks are selected by others.

#### (b) Accounting for the trade‐off in study design

As well as explaining contradictory findings, such as those for the tasty chick hypothesis, identifying quality–vulnerability trade‐offs is important for generating predictions about host choice. A negative correlation can obscure patterns of choice when either quality or vulnerability is considered alone. For example, if brood parasites choose high‐quality hosts, the success of the nests they choose might be expected to be higher than that of randomly experimentally parasitised nests (Soler *et al*., 1995; Langmore & Kilner, 2007). However, as the authors of these studies point out, a negative correlation between quality and vulnerability could lead to a null result instead. This would be the case if cuckoos do choose the highest‐quality hosts, but also experience most rejection from them (such that the success of nests in which parasitic eggs are *found* is no different from the success of experimentally parasitised nests).

### (2) Applying parasite choice to project the effects of anthropogenic change

Anthropogenic change is pervasive and affects a variety of biotic and abiotic features within ecosystems. These include food resource availability, toxins, temperature, and other environmental stressors, each of which can simultaneously affect the quality and vulnerability of hosts (Lafferty & Holt, 2003; Jokela *et al*., 2005). Understanding *how* may be critical to projecting the effects of global change on host–parasite interactions – and consequently, on community‐level dynamics (Budria & Candolin, 2014).

Where the importance of quality exceeds that of vulnerability, we predict environmental stressors that reduce host condition will also reduce parasite success. Conversely, where the importance of vulnerability exceeds that of quality, we predict environmental stressors that reduce host condition will *increase* parasite success.

Food resource availability interacts with both parasite and host fitness (Raffel *et al*., 2008), often mediated *via* host condition (Section II.1.*a*). Reduced food availability for hosts can have positive or negative effects on parasite fitness, depending on how it interacts with both quality and vulnerability (Bittner, Rothhaupt & Ebert, 2002; Ebert *et al*., 2000; Hall *et al*., 2009; Logan, Ruiz‐González & Brown, 2005; Tseng & Myers, 2014). Food availability for many species has been impacted by habitat degradation or destruction, as well as shifting phenology of prey species with temperature changes (Sanz *et al*., 2003; Groom, Meffe & Carroll, 2006; Ockendon *et al*., 2014). Pollution, such as chemical toxins, could also decrease host quality and/or increase host vulnerability to differing degrees. Extreme temperatures may alter parental care provision by nesting birds (Cunningham, Gardner & Martin, 2021), and this could either change in step with behavioural defences against brood parasites, or alter the balance between quality and vulnerability of avian hosts to such a parasite. These two options affect whether parasite choice remains optimal, changes to target different hosts, or lags behind environmental change. Each of these comes with implications for host and parasite populations, either of which may be vulnerable.

With all of the above environmental factors, opposite effects may be seen on individual and population levels. Where stressors disproportionately affect hosts, their increased vulnerability could benefit parasites. However, even if the stressor pushes individual host quality and/or vulnerability in a favourable direction for the parasite, the positive effect may be reversed at the population level. A stressed host population may become smaller and sparser, reducing transmission opportunities for parasites, even if individual vulnerability is higher (Lafferty & Holt, 2003).

### (3) A way of understanding coevolution and co‐adaptation

Antagonistic interactions often lead to coevolution and co‐adaptation (Thompson, 2014). Related adaptations, in particular anti‐parasite defences, can be viewed in light of a quality–vulnerability framework. Hosts evolve adaptions that reduce their vulnerability, or, less commonly, their quality. Physical defences, chemical defences, the immune system, and group living can all function as defences that decrease hosts' vulnerability (Davies *et al*., 2012). Hosts evolving reduced quality is less common, likely because this entails higher fitness costs. Nonetheless, there are potential examples (usually flexibly induced by parasitism). Bacteria can respond to phage infection by depleting their own deoxynucleotides, depriving the phage of an essential DNA component (Tal *et al*., 2022). This may be analogous to hosts reducing their food intake during infection, if this serves to restrict resources available to parasites (Hite, Pfenning & Cressler, 2020). Victims of kleptoparasites can self‐select their own quality and vulnerability *via* their foraging choices: smaller food items tend to have shorter handling times, and therefore reduced quality and vulnerability (Ens, Esselink & Zwarts, 1990).

**Fig. 5:**
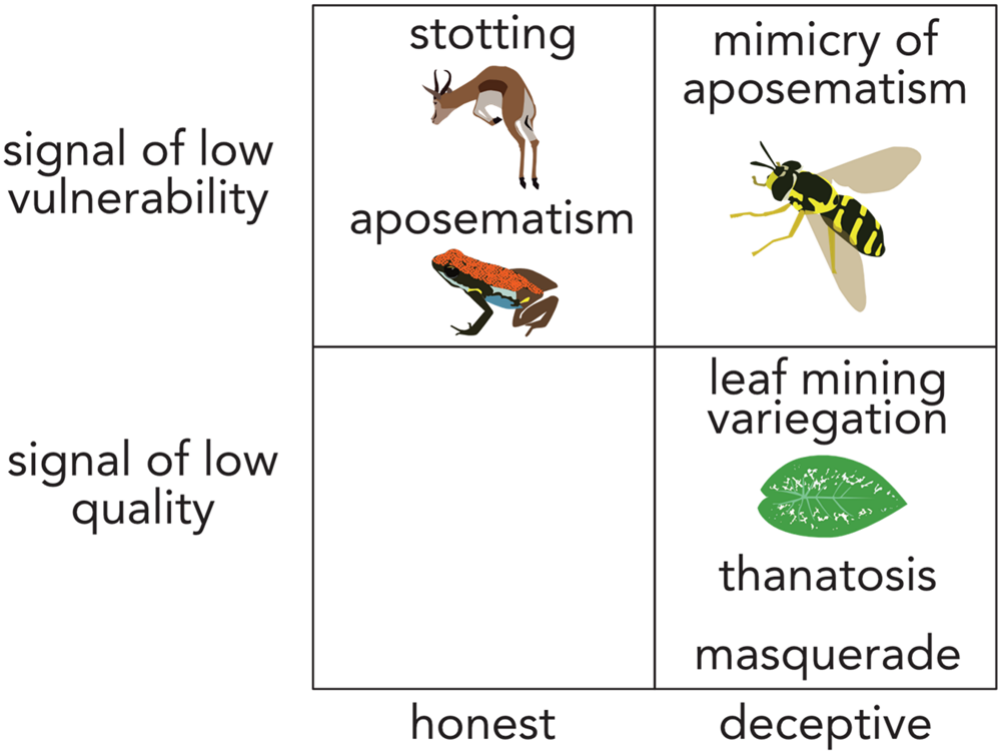
Examples of signals that hosts give in order to reduce the likelihood that a parasite or predator will choose them. Top left: stotting by gazelles signals low vulnerability, as does aposematism (the pairing of a defence with warning colouration or another signal) (FitzGibbon & Fanshawe, 1988; Ruxton *et al*., 2019). Top right: many Batesian mimics deceptively signal low vulnerability by copying the aposematic signals of defended prey (e.g. Pfennig *et al*., 2001). Bottom right: leaf mining variegation is proposed to be a deceptive signal that a leaf is already parasitised, and therefore not worth targeting; prey play dead in thanatosis; and masquerade involves prey resembling inedible objects, such as twigs or bird droppings (Schaefer & Ruxton, 2009; Skelhorn *et al*., 2010; Skelhorn, 2018). Bottom left: honest signals of low quality are less likely than the other options, as these would entail high fitness costs for hosts (if quality for the parasite is similar to quality for the host). While an organism can deceptively signal low vulnerability or genuinely evolve low vulnerability (e.g. aposematism), genuinely evolving low quality is less likely: it is a poor evolutionary strategy actually to be dead or bird droppings [excepting kin‐selected suicide, as seen e.g. in some phage‐infected bacteria (Lopatina *et al*., 2020)].

As well as lowering their quality or vulnerability directly, hosts may manipulate parasite choice by providing deceptive information about these features (Fig. 5). For example, hosts commonly alter cues or signals *via* Batesian mimicry to imply that they are low vulnerability or low quality. For low vulnerability, this ranges from mimicry of chemically defended prey species, to mimicry of a defensive mutualist species (such as ant‐like markings on *Eminium spiculatum* plants) (Bateson, 1892; Jamie, 2017; Lev‐Yadun, 2021).

Hosts can also alter cues of quality to imply they are of low quality (Fig. 5). Thanatosis – in which prey play dead – may be one such example, as already‐dead prey tend to be low in quality (Skelhorn, 2018). Similarly, mimicry of features that suggest prior parasitism could signal low quality by implying resource competition for a prospective parasite. This is evident in mimicry of butterfly eggs or fly larvae tunnels on plant leaves, which have convergently evolved across species, and which deter egg‐laying (thus minimising subsequent insect herbivory (Schaefer & Ruxton, 2009; Soltau, Dötterl & Liede‐Schumann, 2009; Lev‐Yadun, 2021). Deceptive signalling of low quality or low vulnerability has therefore independently evolved across a range of hosts.

The relative importance of host quality *versus* vulnerability to a parasite is likely to affect whether hosts evolve to signal one or the other. This allows us to work backwards, and infer whether quality or vulnerability is more important to a parasite by looking at host signals. Stotting by gazelles may signal low vulnerability to a predator, but potentially also high quality at the same time (FitzGibbon & Fanshawe, 1988). This implies that vulnerability affects predator choice more here than quality (or gazelles would not stot).

Unusually, sometimes prey manipulate predator choice by deceptively signalling *high* vulnerability. This occurs when parents seek to protect their offspring by drawing predators towards themselves instead. Parental birds conspicuously feign injuries in behaviours known as “broken‐wing displays”. These signal high vulnerability and are widespread across bird families in order to distract predators from eggs/chicks (which are genuinely high vulnerability) (de Framond *et al*., 2022).

A trade‐off between quality and vulnerability can also change the anticipated trajectory of mimicry coevolution. For Batesian mimics, increased mimetic accuracy usually reduces attacks by antagonists, and is selected for (Holen & Johnstone, 2018); but this may not be the case if the trait in question is also linked to quality. Body size is one such trait. For predators, attacking prey which are better mimics of a model's body size could prove adaptive if the expected pay‐off from attacks increases disproportionately to the increased risk of making a recognition error (Taylor, 2022). This would not be the case if parasites selected hosts based on vulnerability alone, without reference to quality. Evolution of signalling systems between antagonists can therefore be understood in the context of host quality and vulnerability. Distinguishing between the two is critical, as classic predictions can be reversed depending on whether the model for a Batesian mimic is protected by low quality (profitability) or low vulnerability (harmful defence) (Holen & Sherratt, 2021).

Another broad manifestation of the quality–vulnerability trade‐off could be the evolution of trophic strategies. Taxa in lower trophic levels choose low‐quality, high‐vulnerability “prey” (like grasses), while top predators specialise on high‐quality, low‐vulnerability prey (like antelope). Specialisation may arise from this trade‐off, or initially be determined by other, random factors. Consumers could subsequently evolve adaptations in order to deal with where their prey falls along the quality–vulnerability continuum.

## V. FUTURE RESEARCH DIRECTIONS

Researchers could test directly for the presence of the quality–vulnerability trade‐off in their study systems. Alongside experimental approaches, mathematical models could be used to quantify the trade‐off (Roff & Fairbairn, 2007; Agrawal, 2020). These models could be used to predict host choice by parasites, which might also be tested in empirical systems. Many studies to date present findings that could be explained by either host quality, vulnerability, or both; but studies typically do not test these explanations directly. This is either because studies are correlative, or manipulate an underlying variable (such as food resources or an environmental stressor) which affects both quality and vulnerability simultaneously. In order to test the trade‐off directly, quality and vulnerability need to be experimentally decoupled.

### (1) Challenges for experimental design

This is not trivial in many systems, and may only be feasible in some. Micronutrient supplementation offers one route to reduce host vulnerability without strongly influencing quality, for instance in chick hosts of ectoparasites. This improves immune system functioning with minimal impacts on overall energy resources (Václav & Valera, 2018). This approach has provided clear evidence of the trade‐off's effect: ectoparasitic flies were found generally to select avian European roller (*Coracias garrulus*) hosts on the basis of quality, except at the extremes of each variable (where variation in vulnerability determined parasite choice between hosts of poor body condition, and where hosts with particularly high immunity were avoided). For systems in which front‐line defences determine host vulnerability, experimental infection could isolate the effect of quality on parasite fitness. A key challenge will be to develop methods across different systems that vary one of quality or vulnerability while keeping the other constant.

Several studies use parasitism rates on different biological classes (e.g. ages, sizes or sexes) of host as proxies of their vulnerability (e.g. Poulin, 2013). For instance, high parasitism rates on older age categories are interpreted as evidence that older hosts are more vulnerable. This assumes that parasite choice is primarily based on vulnerability, which – while certainly the case in some systems (see Section II) – may not always be a valid assumption. Host quality has the potential to confound parasitism rates on different types of host and should also be considered when interpreting results. The factors affecting quality and vulnerability can themselves co‐vary, which adds another layer of complexity. Age and size are positively correlated in snails, but appear to affect trematode parasitism in different ways. Older snails experience longer exposure (increasing vulnerability), while larger snails provide a higher quality resource (Gérard & Théron, 1997; Théron, Rognon & Pagès, 1998). To work out which of these factors drives parasite success, they must be decoupled (Graham, 2003).

### (2) Virulence trade‐offs

We may gain additional insights by applying the quality–vulnerability framework to virulence trade‐offs. These are where parasites face a trade‐off between the extent to which they harvest host resources (inflicting harm on the host), and the host's ability to transmit the parasite (Bull, 1994). Higher host quality may minimise this trade‐off, as parasites can extract more resources with less of an effect on the host's ability to transmit them. Meanwhile, high parasite virulence may itself select for host defences, driving the evolution of reduced vulnerability in hosts. Building host quality and vulnerability into concepts of virulence may suggest new avenues for researchers to explore.

### (3) Beyond antagonistic interactions

Finally, future research may extend the framework beyond the antagonistic interactions which are the focus of this review. All interactions that involve a benefit to at least one side involve (*i*) the cost of establishing the interaction with a particular individual, and (*ii*) the benefit accrued from an interaction with that specific individual. This includes mutualisms and commensalisms, which exist on a continuum from antagonism (Ewald, 1987; Bronstein, 1994; Drew, Stevens & King, 2021). Factors (*i*) and (*ii*) may be generalisable to quality and accessibility of the interaction partner. For mutualisms, quality and accessibility may be more likely to co‐vary positively rather than trade off against each other, given the benefit to both partners in establishing the interaction.

## VI. CONCLUSIONS

(1) A generalised trade‐off can characterise host choice by parasites. This trade‐off is between host quality and host vulnerability, and can be applied across different forms of parasitism and predation.
(2) Two conditions are required for a trade‐off between host quality and vulnerability to affect a parasite's choice. The first is the existence of a negative correlation between host quality and vulnerability. This can arise when high host quality selects directly for reduced vulnerability; when reduced vulnerability allows for the evolution of high host quality; or when an independent factor, such as host condition or host number, links the two. The second condition is that parasites must both be capable of, and benefit from, distinguishing between hosts.
(3) Various factors can be used to predict which of quality or vulnerability will have the greater effect on parasite choice. These relate to host and parasite ecology, and include coevolved adaptations of either party. Applying these in the context of global change may help understanding of shifting antagonistic dynamics.
(4) If researchers are able to measure parasite optima, decouple host quality and vulnerability, and share findings across taxa, then we will gain a wider understanding of the mechanisms through which parasites select hosts. This could help to unify research across modes of parasitism and to direct new focuses for research.
